# *Lactiplantibacillus plantarum* ZP-6 mitigates polystyrene nanoplastics-induced liver damage in colitis mice via the gut-liver axis

**DOI:** 10.3389/fmicb.2025.1626941

**Published:** 2025-08-13

**Authors:** Lili Zhao, Zihan Wei, Yibin Wang, Wanrong Chen, Wenjing Zhang, Mengfei Xie, Hong Chen, Yiping Zhang, Haiyan Gao, Xiaobing Jiang

**Affiliations:** ^1^College of Life Sciences, Henan Normal University, Xinxiang, China; ^2^School of Food Science, Henan Institute of Science and Technology, Xinxiang, China; ^3^Henan Engineering Laboratory for Bioconversion Technology of Functional Microbes, College of Life Sciences, Henan Normal University, Xinxiang, China

**Keywords:** *Lactiplantibacillus plantarum*, polystyrene nanoplastic, inflammatory factors, metabonomics, colitic murine, gut-liver axis

## Abstract

**Introduction:**

Nanoplastics (NPs) have become a ubiquitous environmental pollutant that exhibits a tendency to accumulate in large quantities in the tissues of the host body (enteritis patients) with intestinal damage and poses a serious health risk, for which there is currently no suitable method for *in vivo* clearance. Studies have found that lactic acid bacteria has the potential to eliminate pollutants from the body.

**Methods:**

In this study, we investigated the capacity of *Lactiplantibacillus plantarum* ZP-6, a strain isolated from human feces with demonstrated *in vitro* microplastic-binding activity, to alleviate the physiological toxicity of polystyrene nanoplastics (PS-NPs) in healthy and colitic murine models. Then, we investigated the capacity of *Lactiplantibacillus plantarum* ZP-6 to alleviate the physiological toxicity of polystyrene nanoplastics (PS-NPs) in healthy and colitic murine models.

**Results:**

Our findings revealed that PS-NPs exposure resulted in systemic accumulation, triggering organ pathology and inflammatory responses in the liver and colon. Dietary intervention with *Lactiplantibacillus plantarum* ZP-6 significantly reduced PS-NPs retention in blood and tissues while enhancing fecal excretion, restoring hepatic, renal, and colonic histopathology to baseline levels. Mechanistically, ZP-6 downregulated pro-inflammatory cytokines (IL-1β, IL-6, TNF-α) and anti-inflammatory IL-10 in affected tissues. Gut colonization dynamics demonstrated transient enrichment of ZP-6, which facilitated PS-NPs adsorption and fecal clearance. Concurrently, ZP-6 upregulated mucin gene *Muc2* and tight junction components (*OCLN*, *CLDN1*), reinforcing the intestinal epithelial barrier and impeding PS-NPs translocation. Metabolomic analysis further indicated that ZP-6 rectified PS-NPs-induced hepatic metabolic dysregulation via the gut-liver axis.

**Conclusion:**

These results elucidate a multifaceted probiotic mechanism for NPs detoxification, providing a promising translational strategy to counteract nanoplastic-related health hazards.

## 1 Introduction

Nanoplastics (NPs), defined as plastic particles with diameters below 100 nm (Mitrano et al., 2021), have become ubiquitous due to the extensive use of plastic products. These particles are readily ingested by organisms and have now been detected in a wide range of species, including humans (Schwabl et al., 2019). Substantial animal studies demonstrate that NPs can disrupt intestinal epithelial permeability, trigger gut inflammation, impair normal intestinal function, and even promote the development of chronic diseases (Hirt and Body-Malapel, 2020). In toxicological studies of marine organisms, NPs exposure has been shown to reduce digestive enzyme secretion, induce hepatic lipid metabolism disorders (Gonzalez-Fernandez et al., 2021; Lai et al., 2021; Li et al., 2021), upregulate antioxidant gene expression, cause immune dysregulation, and suppress muscle growth (Brandts et al., 2021), ultimately decreasing survival rates. Notably, polystyrene nanoplastics (PS-NPs) accumulate in the gut, reducing mucus secretion and compromising intestinal barrier integrity (Jin et al., 2019). Deng et al. (2017) detected NPs in mouse tissues, with significant accumulation observed in the liver, kidneys, and intestines. Metabolomic analyses further reveal that NPs exposure alters hepatic metabolic profiles in fish, disrupting lipid and energy metabolism (Lu et al., 2016). While growing concerns over the adverse effects of NPs have spurred increasing research into their toxicity mechanisms, few studies have explored potential defense strategies against NPs-induced damage. Consequently, there is an urgent need to identify effective approaches to mitigate NPs toxicity.

Inflammatory bowel disease (IBD), a chronic inflammatory disorder encompassing ulcerative colitis (UC) and Crohn’s disease, has rising global prevalence. While Crohn’s disease involves transmural inflammation across the gastrointestinal tract, UC specifically targets the colonic mucosa — making it pathologically congruent with experimental colitis models (Xavier & Podolsky, 2007). This UC pathology is characterized by diffuse inflammation and epithelial barrier disruption in the colon, which may facilitate systemic translocation of environmental contaminants (Sandys and Te Velde, 2022). NPs, pervasive foodborne pollutants, accumulate in the gut and exhibit enhanced penetration through the compromised intestinal barrier in UC/colitis (Pironti et al., 2021). Notably, the increasing environmental burden of plastic particles correlates with IBD incidence trends, particularly UC (Molodecky et al., 2012). Epidemiological evidence from Taiwan, China reveals that in regions with high consumption of seafood from polluted waters, UC incidence surged 1.5-fold (1998-2008) (Tong et al., 2013). Critically, Yan et al. (2022) demonstrated elevated NPs in feces of IBD patients, with the highest particle loads observed in UC subgroups—positively correlating with disease severity. These findings establish a focused research imperative: UC-associated colitis creates a “leaky gut” permissive to NPs translocation, potentially driving extra-intestinal damage via portals like the gut-liver axis.

The gut and liver maintain intricate physiological and pathological connections through the “gut-liver axis.” Gut microbiota-derived products, including bioactive metabolites and microbe-associated molecular patterns, can traverse the intestinal barrier, enter the portal circulation, and subsequently modulate hepatic pathophysiology during disease progression (Lin et al., 2025). The intestinal barrier serves as a crucial defense system that prevents the translocation of harmful substances such as toxins into systemic tissues and microcirculation, pathological conditions including trauma, stress, and inflammatory responses can compromise intestinal barrier integrity to varying degrees, thereby exacerbating primary disease conditions (Allaire et al., 2018). As the primary detoxification organ, the liver is continuously exposed to numerous endogenous and exogenous toxins that may induce hepatic damage. Emerging evidence suggests that maintaining gut microbial symbiosis may enhance hepatic anti-inflammatory and antioxidant responses, consequently protecting against acute hepatotoxicity. For instance, probiotic administration has been demonstrated to ameliorate aflatoxin B1 (AFB1)-induced toxicity in humans (Mohd Redzwan et al., 2016). These findings highlight the pivotal role of gut microbiota and intestinal homeostasis in modulating toxin-mediated acute liver injury.

*Lactiplantibacillus plantarum* (*L. plantarum*), a prominent probiotic species within the gut microbiota, is ubiquitously present in various food products and the gastrointestinal tract (Nordström et al., 2021). Accumulating evidence demonstrates its pivotal role in mitigating the detrimental effects of food contaminants on host health. Specifically, *L. plantarum* facilitates heavy metal excretion and reduces tissue accumulation, thereby attenuating metal toxicity. The protective mechanisms include preservation of intestinal barrier integrity and modulation of oxidative stress to inhibit intestinal heavy metal absorption (Duan et al., 2020). *In vivo* murine studies reveal that *L. plantarum* CCFM8610 significantly decreases cadmium accumulation in cadmium-exposed mice while enhancing fecal cadmium elimination (Zhai et al., 2013). Furthermore, *L. plantarum* exhibits multi-faceted therapeutic potential in inflammatory bowel disease through: regulation of inflammatory cytokines; potentiation of anti-inflammatory factor secretion; amelioration of intestinal inflammation (Mishima et al., 2019); Notably, oral administration of *L. plantarum* 06CC2 stimulates IL-10 production in the colon, alleviating dextran sulfate sodium (DSS)-induced colitis in mice (Tanaka et al., 2020). Similarly, *L. plantarum* CBTLP3 restores goblet cell populations and suppresses pro-inflammatory cytokine secretion in DSS-induced colitis models (Kim et al., 2020). Building upon preliminary findings that *L. plantarum* ZP-6 (isolated from human feces) exhibits nanoplastic clearance capacity *in vitro*, we subsequently investigated its therapeutic potential for eliminating NPs *in vivo* using murine models.

Given the escalating burden of plastic pollution, PS-NPs serve as a model to investigate NPs toxicity and remediation. Building upon our previous isolation of *L. plantarum*—a human fecal strain demonstrating *in vitro* NPs adsorption capacity—this study aimed to quantify the effect of ZP-6 on *in vivo* fecal PS-NPs excretion, assess its ability to restore PS-NPs-compromised intestinal barrier integrity, evaluate its anti-inflammatory effects against systemic inflammation, and examine its capacity to rectify PS-NPs-induced metabolic dysregulation via the gut-liver axis. Here, we hypothesize that ZP-6 mitigates PS-NPs-induced damage by: (i) enhancing fecal NP excretion, (ii) restoring gut barrier integrity, (iii) modulating inflammation, and (iv) rectifying metabolic dysregulation via the gut-liver axis. By testing this hypothesis in healthy and colitic mice, we aim to develop a probiotic-based dietary strategy to counteract NPs toxicity.

## 2 Materials and methods

### 2.1 Tested strains and materials

The study utilized *L. plantarum* (strain ZP-6), previously isolated from infant feces in our laboratory. Commercial 100 nm polystyrene microspheres were obtained from Dongguan Mingyuxing Plastic Raw Materials Co., Ltd. Five-week-old male mice were supplied by Henan Xincheng Youkang Biotechnology Co., Ltd. The animals were housed under controlled conditions: temperature maintained at 23 ± 1°C, relative humidity at 50% ± 5%, with natural light cycles. Mice had ad libitum access to food and water throughout the study period.

### 2.2 Preparation of fluorescent PS-NPs

Nile red dye (0.01 mg) was dissolved in chloroform (1 mL) to prepare the Nile red dye solution (0.01 mg/mL) that was kept in dark. Separately, PS-NPs solution was made by dispersing 250 mg of PS-NPs in water-DMSO (v:v = 1:1) mixture. The Nile red dye solution was then added to the PS-NPs dispersion, and the mixture was heated at 75°C for 30 min. Immediately after heating, the solution was rapidly cooled in an ice-water bath. The cooled suspension was filtered through a polycarbonate membrane (30 nm pore size), after which the retained PS-NPs were transferred to pure water and stored protected from light.

### 2.3 Animal experimental methods

**Figure 1:**
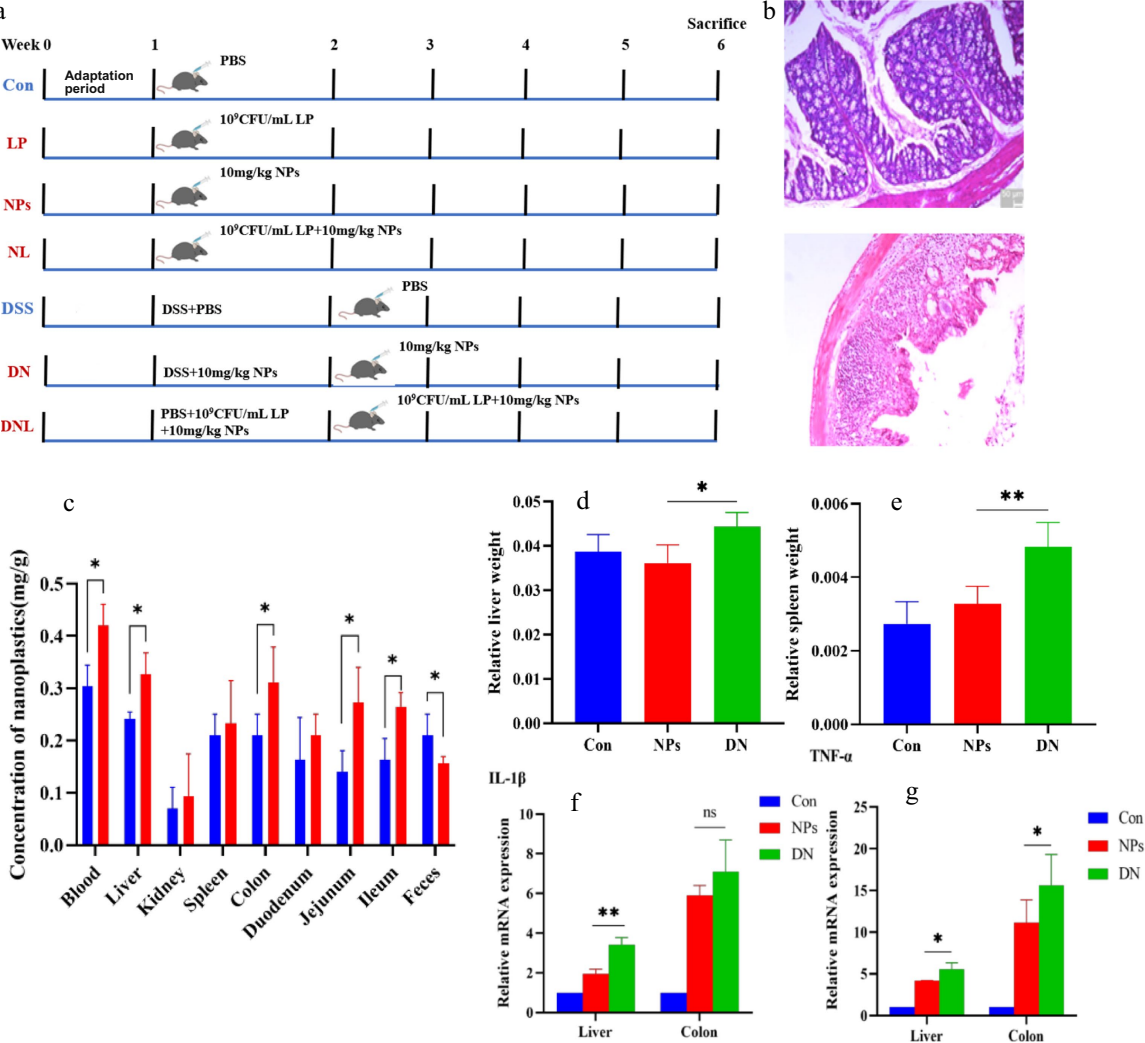
**(a)** Experimental design. Con—control group; LP—*L. plantarum* group; NPs—nanoplastic group; NL—nanoplastic + *L. plantarum* group; DSS—acute colitis group; DN—colitis + nanoplastic group; DNL—colitis + nanoplastic + *L. plantarum* group. **(b)** Colon pathological sections of health mice and mice with colitis. **(c)** Content of PS-NPs in mouse tissues. **(d,e)** Changes in mice body weight and organ coefficients. **(f,g)** Expression of inflammatory factors in mouse liver and colon. ns express *p* > 0.05, *express *p* < 0.05, **express *p* < 0.01, *n* = 5.

Animal experiments were approved by the Animal Ethics Committee of Henan Normal University, China. Twenty 5-week-old male SPF grade C57BL/6 mice were purchased from Henan Xincheng Youkang Biotechnology Co., Ltd. The mice underwent a 1-week adaptation period under free access to food and water. Subsequently, the mice were divided into a healthy group and a colitis model group (Figure 1a). The healthy group consisted of four groups (*n* = 5 in each group): control group (Con): 0.2 mL PBS was administered orally daily; *L. plantarum* ZP-6 group (LP): 0.2 mL *L. plantarum* ZP-6 bacterial solution was administered orally daily (5 × 10^9^ CFU/mL, the dose of *L. plantarum* ZP-6 was determined by the previous research of the research group); PS-NPs group (NPs): 0.2 mL fluorescent PS-NPs (10 mg/kg BW) were administered by gavage every day; PS-NPs + *L. plantarum* ZP-6 group (NL): 0.2 mL fluorescent PS-NPs + 0.2 mL *L. plantarum* ZP-6 solution were intragastrically administered every day.

Colitis model group consisted of three groups (*n* = 5 in each group): ① colitis group (dextran sulfate sodium, DSS): refer to previous studies for the modeling method of acute colitis mice. Specifically, mice were free to drink water containing 3% DSS (w/v; mw36,000–50,000) for 7 days, provided with maintenance feed for mice, and gavaged with 0.2 mL PBS every day; ② Colitis + PS NPs group (DN): on the basis of drinking 3% DSS, 0.2 mL fluorescent PS-NPs (10 mg/kg BW) was administered by gavage every day; ③ Colitis + PS NPs + *L. plantarum* ZP-6 group (DNL): on the basis of drinking 3% DSS, mice were given 0.2 mL of fluorescent PS-NPs (10 mg/kg BW) + *L. plantarum* ZP-6 solution by gavage every day.

The dose of *L. plantarum* ZP-6 was determined as the optimal concentration through previous research and exploration by our research team. The experimental period was 5 weeks. At the end of the 5th week, the mice were anesthetized with isoflurane, and blood was collected from the orbit. The mice were euthanized by cervical dislocation. The liver, kidney, spleen, colon and small intestine were removed, part of which was fixed with 4% paraformaldehyde, and the rest was quickly frozen with liquid nitrogen and stored in a −80°C refrigerator.

### 2.4 Determination of body weight and organ coefficient of mice

The weight of the mice was measured before the experiment, and the weight of the mice was recorded every 7 days after the start of intragastric administration. The weight of the mice was measured before the mice were killed. After the mice were dissected, the liver, spleen and kidney were collected, weighed, and divided by the weight of the mice to obtain the organ coefficient. The length of the small intestine and colon were measured respectively.

### 2.5 Distribution of PS-NPs in mice

Mouse liver, kidney, spleen, small intestine, and colon were fixed, dehydrated, and embedded. Tissue sections (5 μm) were prepared using a Leica CM1860 cryostat. Sections were examined under a fluorescence microscope (Leica DMIL Microsystems) equipped with: TRITC filter (Excitation: 550/20 nm; Emission: 630/40 nm), 20× objective and 10× eyepiece (Total magnification: 200×).

### 2.6 Content of PS-NPs in organs, blood and fecal tissues

After intragastric administration of fluorescent PS-NPs in mice, the blood of mice in NPs group and NL group was obtained by orbital blood collection at 0, 4, 8, 12 and 24 h, and the blood of mice in DN group and DNL group was collected at 3, 5, 7, 9, 11 and 13 h after the start of modeling. The blood volume was 200 μL, and the feces of mice were collected at the same time point. Blood, liver, kidney, spleen, small intestine, colon and fecal tissues were collected after 5 weeks of intragastric administration. Approximately 0.2 g of sample was dissolved in a mixture of HNO₃ and H₂O₂ until no visible solid residue remained, followed by dilution to volume with deionized water. The fluorescence value of the sample was measured at the excitation/emission wavelength of 543 nm/620 nm using a microplate reader (Agilent Technologies SH1M2F). The standard curve was obtained by a series of concentrations of fluorescent PS-NPs (0, 0.1, 0.2, 0.4, 0.6, 0.8 mg/mL). The value of PS-NPs level in the sample was obtained by offsetting the value of the control group. The fluorescence value of the control group (representing untreated sample) was subtracted from the sample fluorescence value to correct for background interference. The PS-NPs concentration in the sample was then determined by interpolating this corrected fluorescence value onto the standard curve.

### 2.7 Histopathological analysis

The intact liver, kidney, spleen and colon tissues of mice were fixed in 4% paraformaldehyde for 24 h at room temperature. Fixed tissues were then dehydrated through a graded ethanol series (70–100%, 35 min per concentration). Following dehydration, samples were cleared using sequential xylene incubations: first in a 1:1 (v/v) ethanol-xylene mixture for 10 min, followed by two changes of pure xylene. Tissue infiltration was performed with molten paraffin wax at 65°C. Organs were subsequently embedded in paraffin blocks, solidified at −20°C. Serial sections of 5 μm thickness were cut using a Leica CM1860 microtome. The tissue sections were stained with Hematoxylin and Eosin (H&E) Staining, and permanently mounted with neutral balsam under coverslips. Stained tissue sections were visualized using an OLYMPUS BX43 brightfield microscope for comprehensive histopathological assessment.

### 2.8 Expression analysis of inflammatory factors and intestinal barrier related factors

The fresh tissues of liver, colon and duodenum of mice were ground and added with RNA extract to extract total RNA, and the RNA concentration was determined by micro spectrophotometer. Reverse transcription was performed according to the instructions of the cDNA reverse transcription kit. The expression of *IL-1β* (Interleukin-1β), *IL-6* (Interleukin-6), *IL-10* (Interleukin-10), *TNF-α* (Tumor Necrosis Factor-α) and intestinal barrier related factors *OCLN* (Occludin), *CLDN1* (Claudin 1), *Muc2* (Mucin 2) were detected by fluorescent quantitative PCR using cDNA as template.

#### 2.8.1 RNA extraction and quantification

Fresh tissues (liver, colon, duodenum; 0.1 g each) were homogenized in 200 μL RNA lysis buffer on ice using a tissue grinder, followed by addition of 800 μL lysis buffer and centrifugation (12,000 rpm, 4°C, 10 min). The supernatant was transferred to a new tube, mixed with chloroform (20% v/v), vortexed for 15 s, incubated for 5 min, and centrifuged (12,000 rpm, 4°C, 15 min). The aqueous phase was collected, combined with an equal volume of isopropanol, inverted to mix, incubated for 5 min, and centrifuged (12,000 rpm, 4°C, 10 min). After discarding the supernatant, the RNA pellet was washed with 1 mL 75% ethanol (7,500 × g, 4°C, 5 min), air-dried, and dissolved in 30 μL DEPC-H₂O. RNA concentration was determined using a Nanodrop ND-2000 spectrophotometer (Thermo Fisher) with DEPC-H₂O as blank and 1 μL sample loading volume.

#### 2.8.2 cDNA synthesis via reverse transcription

Genomic DNA was removed from RNA templates (1 μg) using a 10 μL reaction containing 1 μL 10 × gDNA remover mix in nuclease-free H₂O. Reactions were incubated at 42°C for 2 min (metal bath), then immediately cooled on ice. Reverse transcription was performed by adding 4 μL 5 × M5 RT Super Plus Mix and 6 μL nuclease-free H₂O to the 10 μL gDNA-cleared RNA, yielding a 20 μL total volume. After gentle mixing and brief centrifugation, samples were incubated at 37°C for 15 min followed by enzyme inactivation at 85°C for 5 s. Synthesized cDNA was stored at −20°C.

#### 2.8.3 Real-time PCR

The primers were synthesized by Shanghai Sanggong Bioengineering Co., Ltd., and the specific sequences are shown in Table 1. Real-time PCR reaction system: 10 μL 2 × SGExcel FastSYBR Mixture; 0.4 μL each forward/reverse primer (10 μM); 5 μL cDNA template; Nuclease-free water to 20 μL final volume. qPCR was performed using SYBR Green Master Mix (Applied Biosystems) with primers validated against Primer Bank sequences. Amplification conditions: 95°C (3 min); 40 cycles of 95°C (5 s)/60°C (20 s); melt curve analysis. Relative expression was normalized to β-actin using the 2^−ΔΔCT^ method. Amplicon sizes were confirmed by agarose gel electrophoresis. Primer efficiencies (98–102%) were determined from standard curves (*R*^2^ > 0.99) using serial cDNA dilutions.

**Table 1:** Primer sequences and amplification parameters.

Genes	Direction	Sequence (5′ → 3′)	Product length (bp)	Annealing temperature (°C)	Efficiency (%)
*IL-1β*	Forward	GAAATGCCACCTTTTGACAGTG	116	58	101
	Reverse	TGGATGCTCTCATCAGGACAG			
*IL-6*	Forward	CTGCAAGAGACTTCCATCCAG	131	57	103
	Reverse	AGTGGTATAGACAGGTCTGTTGG			
*IL-10*	Forward	CTTACTGACTGGCATGAGGATCA	101	58	103
	Reverse	GCAGCTCTAGGAGCATGTGG			
*TNF-α*	Forward	CCTGTAGCCCACGTCGTAG	148	59	101
	Reverse	GGGAGTAGACAAGGTACAACCC			
*β-actin*	Forward	GGGAAATCGTGCGTGAC	176	55	105
	Reverse	AGGCTGGAAAAGAGCCT			
*OCLN*	Forward	TGAAAGTCCACCTCCTTACAGA	128	55	102
	Reverse	CCGGATAAAAAGAGTACGCTGG			
*CLDN1*	Forward	GCCTTGATGGTAATTGGCATCC	165	58	105
	Reverse	GGCCACTAATGTCGCCAGAC			
*Muc2*	Forward	AGGGCTCGGAACTCCAGAAA	106	59	102
	Reverse	CCAGGGAATCGGTAGACATCG			

### 2.9 Analysis of intestinal colonization of *L. plantarum* ZP-6

FITC (fluorescein isothiocyanate) solid powder was mixed in dimethyl sulfoxide to form a staining solution with a concentration of 0.1 mg/mL, and then added to the suspension of *L. plantarum* ZP-6 for staining. *L. plantarum* ZP-6 (200 μL) was fed into mice by gavage, and six time gradients of 0, 2, 4, 8, 12, and 24 h were set. At these six time points, the mice were euthanized, the colon tissue was taken, cut and spread on the slide, and then the existence of *L. plantarum* ZP-6 in the colon was observed under the inverted fluorescence microscope.

### 2.10 Analysis of PS-NPs adsorption by *L. plantarum* ZP-6

Mice were intragastrically administered with fluorescent PS-NPs and *L. plantarum* ZP-6 2 h later. Feces were collected within 2 h after intragastric administration of *L. plantarum* ZP-6. The feces were prepared into fecal suspension, and the upper turbid liquid smear was taken to observe the fluorescence under the laser scanning confocal microscope.

### 2.11 Analysis of intestinal mucus secretion

The method provided by AB-PAS (Alcian Blue-Periodic Acid Schiff) staining kit was used to stain the colonic tissue sections of mice. After staining, the secretion of colonic mucus in each group was observed under the microscope, and the area of the mucus part was quantified by ImageJ software to obtain the mucus coverage rate for comparison.

### 2.12 Hepatic metabonomic analysis

Fifty milligrams of liver and pre cooled mixture (methanol: acetonitrile: water = 2:2:2, v/v/v) were treated with vortex and ultrasound, incubated at −20°C for 10 min, and then centrifuged for 20 min. The supernatant was dried under vacuum, redissolved in 600 μL solvent (acetonitrile: water = 1:1, v/v) and centrifuged. Then the supernatant was collected for subsequent analysis. The metabolites were quantified by Thermo Fisher vanquish ultra performance liquid chromatography-q active focus electrostatic field orbital trap mass spectrometry (LC-MS/MS). Metaboanalyst 6.0 was used for metabolomic data analysis (Fu et al., 2022). Partial least squares discriminant analysis (PLS-DA) was performed to visualize the metabolic differences between the experimental groups after mean centralization and unit variance scaling (Zhao et al., 2019).

### 2.13 Data processing and analysis

Data are expressed as mean ± standard error. At least 3 replicates shall be set for each test. Graphpad prism 8.4.0 (LA Jolla, CA, United States) was used for data analysis. Tukey test was used for one-way analysis of variance (ANOVA). When *p* < 0.05, the results were considered statistically significant.

## 3 Results

### 3.1 Impact of PS-NPs on colitis progression in murine models

Unlike previous studies that administered NPs after colitis onset, this investigation employed a concurrent exposure model where PS-NPs were administered via intragastric gavage simultaneously with DSS-induced colitis modeling. This experimental paradigm allows for comprehensive evaluation of NP effects during: (i) the pre-inflammatory phase, (ii) acute colitis manifestation, and (iii) the subsequent recovery period. Importantly, this approach better recapitulates real-world scenarios where environmental NP exposure coincides with colitis development in human patients, thereby enhancing the clinical relevance of our findings. DSS-induced colitis, characterized by pronounced epithelial damage, crypt loss, inflammatory cell infiltration, and mucosal thickening (Figure 1b), was successfully established as evidenced by histopathological confirmation. To assess the potential exacerbating effects of PS-NPs on colitis progression, we conducted comparative analyses between healthy controls (NP_S_ group) and PS-NP-treated colitis mice (DN group).

The results revealed that PS-NPs exposure significantly (*p* < 0.05) increased: hepatosplenic indices (liver and spleen coefficients) in colitis mice compared to healthy controls; PS-NPs accumulation in systemic circulation (blood) and target organs (liver, colon, jejunum, and ileum); pro-inflammatory cytokine levels in colitis-affected animals (Figures 1c–g). These data collectively demonstrate that PS-NPs exert more pronounced pathological effects in the context of intestinal inflammation, potentially through enhanced tissue accumulation and amplified inflammatory responses. The observed organ coefficient changes suggest possible systemic involvement beyond the gastrointestinal tract.

### 3.2 *L. plantarum* ZP-6 modulates systemic distribution and fecal excretion of PS-NPs in mice

#### 3.2.1 Systemic circulation analysis

**Figure 2:**
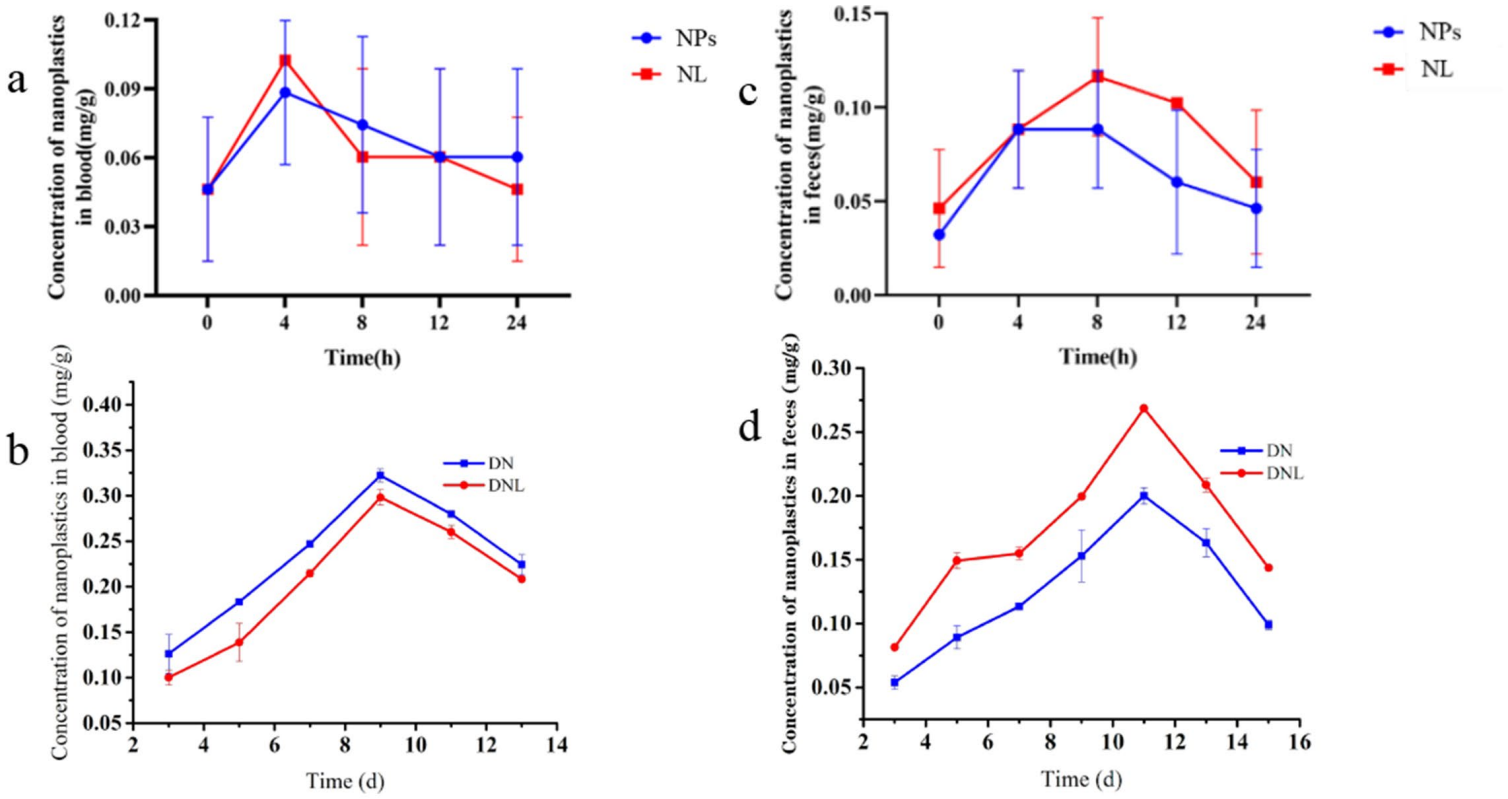
**(a,b)** Changes in nanoplastic content of mice blood. **(c,d)** Changes in nanoplastic content of mice feces. Con—control group; LP—*L. plantarum* group; NPs—nanoplastic group; NL—nanoplastic + *L. plantarum* group; DSS—acute colitis group; DN—colitis + nanoplastic group; DNL—colitis + nanoplastic + *L. plantarum* group; ns express *p* > 0.05, *express *p* < 0.05, **express *p* < 0.01, ***express *p* < 0.001, *n* = 5.

To investigate the pharmacokinetics of orally administered polystyrene nanoparticles (PS-NPs), we quantified their temporal distribution in blood and feces during the first 24 h post-administration (Figure 2). Pharmacokinetic analysis revealed rapid absorption of PS-NPs, with blood concentration peaking at 4 h (0.0884 ± 0.0315 mg/g) followed by gradual decline, suggesting subsequent tissue redistribution (Figure 2a). Notably, prophylactic administration of *L. plantarum* ZP-6 (initiated 4 h post-PS-NPs exposure) significantly attenuated systemic PS-NPs accumulation compared to NPs-only controls (*p* < 0.05, Figure 2b).

In DSS-induced colitis models (DN group), we observed exacerbated PS-NPs absorption kinetics, with peak blood concentrations (0.3224 ± 0.0700 mg/g) coinciding with maximal disease severity on day 9 (Figure 2b). This temporal correlation suggests compromised intestinal barrier integrity facilitates enhanced systemic nanoparticle translocation. The subsequent decline in circulating PS-NPs after day 10 paralleled clinical improvement (body weight recovery, reduced hematochezia), while *L. plantarum* ZP-6 supplementation (DNL group) showed limited efficacy during active inflammation, indicating gut barrier integrity modulates probiotic-mediated nanoparticle clearance.

#### 3.2.2 Fecal excretion dynamics

The pharmacokinetic analysis revealed distinct patterns of fecal PS-NPs excretion contingent upon intestinal health status. In healthy mice, PS-NPs excretion followed a biphasic kinetic profile, characterized by a sharp peak (0.0883 ± 0.0372 mg/g) within 8 h post-exposure, followed by a subsequent decline (Figure 2c). Critically, administration of *L. plantarum* ZP-6 significantly enhanced the magnitude of fecal PS-NPs elimination throughout this excretion timeline without altering its biphasic nature. Conversely, in mice with colitis, the profound impairment of intestinal barrier function severely compromised baseline PS-NPs excretion capacity. While probiotic intervention with ZP-6 significantly increased excretion compared to untreated colitic mice, it achieved only partial restoration, failing to reach the efficiency observed in healthy animals (Figure 2d).

These findings collectively demonstrate that PS-NPs undergo rapid enteric absorption (peak plasma concentration at 4 h) and systemic distribution. The efficacy of *L. plantarum* ZP-6 in enhancing fecal PS-NPs clearance is evident in both healthy and inflamed intestines; however, its therapeutic potential is markedly constrained under conditions of colitis. This differential efficacy directly underscores the paramount importance of gut barrier integrity as a critical determinant governing not only systemic PS-NPs burden but also the success of probiotic-mediated detoxification strategies. Consequently, while *L. plantarum* ZP-6 represents a promising intervention for promoting nanoplastic elimination via the fecal route, its detoxification capacity is fundamentally contingent upon the functional status of the intestinal barrier. These results highlight the gut barrier’s pivotal role in modulating nanoplastic biodistribution and solidify the rationale for developing probiotic-based approaches to mitigate environmental toxicant exposure, particularly emphasizing the need to consider underlying gut health.

### 3.3 Effect of *L. plantarum* ZP-6 on the tissue distribution and accumulation of PS-NPs in mice

#### 3.3.1 Tissue distribution analysis

**Figure 3:**
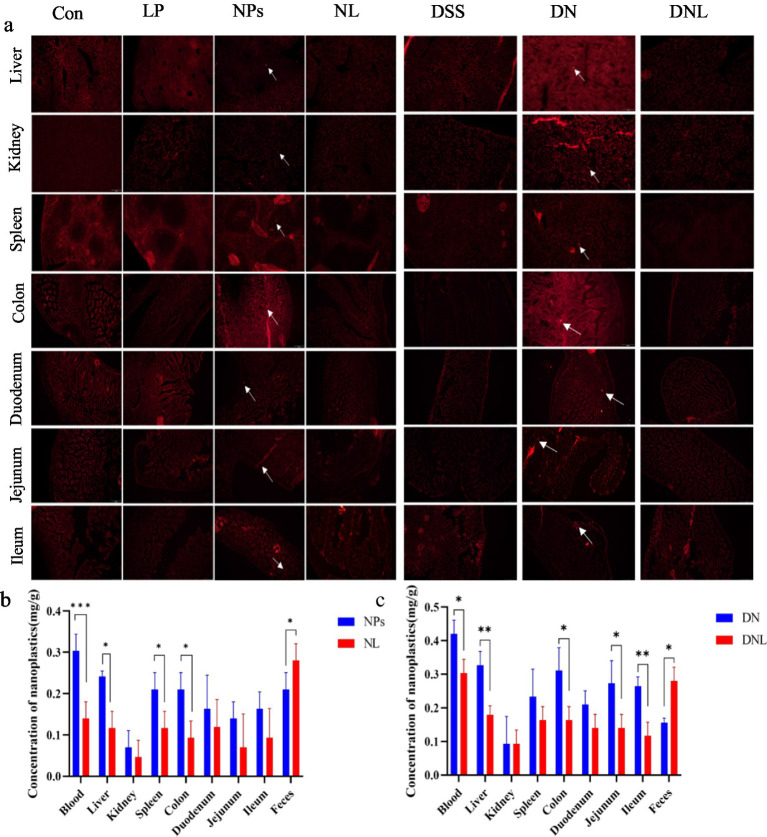
**(a)** Distribution of nanoplastics in various organs of mice. **(b,c)** Content of PS-NPs in mouse tissues. Con—control group; LP—*L. plantarum* group; NPs—nanoplastic group; NL—nanoplastic + *L. plantarum* group; DSS—acute colitis group; DN—colitis + nanoplastic group; DNL—colitis + nanoplastic + *L. plantarum* group; *express *p* < 0.05, **express *p* < 0.01, ***express *p* < 0.001, *n* = 5.

To assess the biodistribution of polystyrene nanoparticles (PS-NPs) in murine tissues, frozen sections of the liver, kidney, spleen, colon, duodenum, jejunum, and ileum were examined via fluorescence microscopy. As depicted in Figure 3a, fluorescent PS-NPs were widely distributed across multiple organs following oral administration. However, treatment with *L. plantarum* ZP-6 significantly attenuated PS-NPs accumulation in these tissues. In the NL group (healthy mice administered *L. plantarum* ZP-6), fluorescent PS-NPs were nearly undetectable in all organs except the ileum. Conversely, in the DNL group (DSS-induced colitis mice treated with *L. plantarum* ZP-6), residual fluorescence was observed in various tissues, particularly in intestinal segments, though at markedly lower intensities compared to the DN group (DSS-induced colitis mice without probiotic intervention). These findings demonstrate that *L. plantarum* ZP-6 effectively mitigates PS-NPs retention in systemic organs.

#### 3.3.2 Quantitative analysis of PS-NPs accumulation

To quantify PS-NPs burdens, tissues (blood, liver, kidney, spleen, colon, duodenum, jejunum, ileum) and fecal samples were collected, digested, and analyzed via fluorometry using a calibrated standard curve (*y* = 14.266*x* − 0.4608, *R*^2^ = 0.9972). In healthy mice (Figure 3b), PS-NPs predominantly accumulated in the circulatory system (highest concentration), followed by hepatic tissue. This distribution pattern suggests that ingested PS-NPs undergo transepithelial uptake in the gastrointestinal tract, entering systemic circulation as nanoparticulate fractions. The liver, while serving as the primary metabolic organ, exhibited significant PS-NPs retention due to its filtration capacity and inability to metabolize synthetic polymers. Colonic accumulation likely reflects prolonged transit time, with partial PS-NPs redistribution into circulation via enterocyte uptake, fecal excretion, or luminal retention.

Notably, *L. plantarum* ZP-6 intervention (NL group) significantly reduced PS-NPs loads in blood, liver, spleen, and colon while enhancing fecal elimination (*p* < 0.05 vs. NP_S_ controls). In colitis models (Figure 3c), the DNL group showed pronounced reductions in PS-NPs content in blood, liver, colon, and small intestinal segments compared to DN controls (*p* < 0.05), alongside increased fecal excretion. Collectively, these data indicate that *L. plantarum* ZP-6 enhances PS-NPs clearance via the fecal route and reduces systemic bioaccumulation.

### 3.4 *L. plantarum* ZP-6 attenuates PS-NPs-induced organ damage in mice

#### 3.4.1 Restoration of intestinal morphology

**Figure 4:**
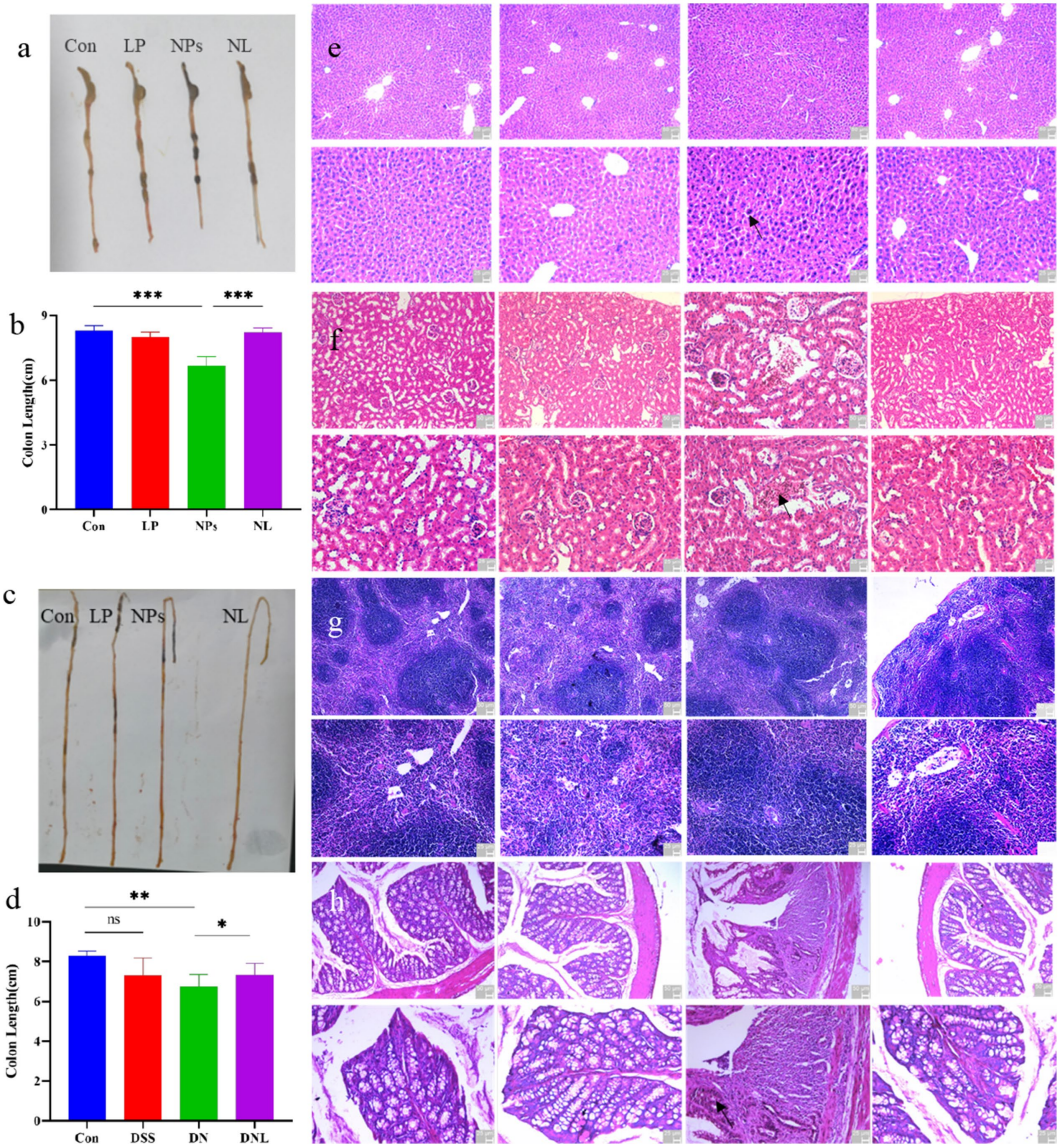
**(a–d)** Changes in mice colon and small intestinal morphology. The organ status of mice **(e)** liver; **(f)** kidney **(g)** spleen; **(h)** colon. Con—control group; LP—*L. plantarum* group; NPs—nanoplastic group; NL—nanoplastic + *L. plantarum* group; DSS—acute colitis group; DN—colitis + nanoplastic group; DNL—colitis + nanoplastic + *L. plantarum* group; *express *p* < 0.05, **express *p* < 0.01, ***express *p* < 0.001, *n* = 5.

The gastrointestinal tract serves as the primary target for PS-NPs accumulation, with intestinal length serving as a sensitive biomarker of inflammatory severity. As illustrated in Figures 4a,b, chronic (5-week) PS-NPs exposure induced significant colonic shortening (*p* < 0.05 vs. control), a hallmark of intestinal inflammation. Strikingly, *L. plantarum* ZP-6 supplementation (NL group) restored colonic length to near-baseline levels, demonstrating therapeutic efficacy.

Unexpectedly, small intestinal morphology exhibited divergent responses (Figure 4c). While PS-NPs exposure (NPs group) triggered significant elongation of the small intestine (*p* < 0.05)—suggesting compensatory hyperplasia or structural remodeling—*L. plantarum* ZP-6 failed to normalize this parameter. These findings indicate that PS-NPs differentially disrupt intestinal homeostasis, with *L. plantarum* ZP-6 selectively mitigating colonic but not small intestinal pathology.

In DSS-induced colitis models, PS-NPs exacerbated disease progression. The DN group (DSS + PS-NPs) exhibited persistent colonic shortening (1.50 cm reduction vs. control, Figure 4d), contrasting with the self-resolving pathology in DSS-only mice. Notably, *L. plantarum* ZP-6 intervention (NDL group) partially reversed this atrophy (*p* < 0.05 vs. DN), underscoring its protective role in inflammatory settings.

#### 3.4.2 Histopathological assessment of systemic organ damage

H&E-stained paraffin sections of major organs revealed distinct PS-NPs-induced pathologies: *Liver*: Parenchymal cell hyperchromasia and nuclear swelling (Figure 4a), indicative of hepatocellular stress (Figure 4e); *Kidney*: Proximal tubule dilation and peritubular inflammatory infiltrates (Figure 4f), suggesting acute tubular injury; *Spleen*: No significant microarchitectural alterations (Figure 4g); *Colon*: Crypt distortion, villus blunting, and transmural leukocyte infiltration (Figure 4h), confirming severe colitis. Remarkably, *L. plantarum* ZP-6 treatment (NL group) preserved hepatic, renal, and colonic histoarchitecture at levels indistinguishable from healthy controls. This systemic protection highlights the strain’s capacity to counteract PS-NPs toxicity across multiple organ systems.

### 3.5 Mechanistic insights into *L. plantarum* ZP-6-mediated microplastic clearance

#### 3.5.1 Intestinal colonization dynamics of *L. plantarum* ZP-6

**Figure 5:**
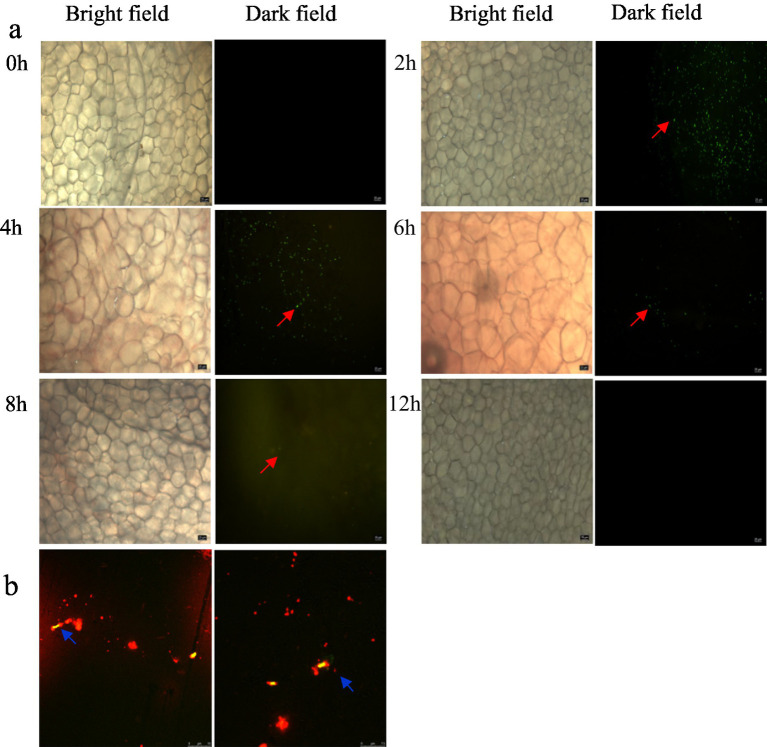
**(a)** Analysis of the colonization of *L. plantarum* ZP-6 in the intestinal tract of mice. **(b)** Combination of *L. plantarum* ZP-6 and nanoplastics in mice feces. Those that emit red fluorescence are PS-NPs, and those that emit yellow-green fluorescence are *L. plantarum* ZP-6.

To elucidate the PS-NPs clearance mechanism, we tracked the intestinal colonization pattern of FITC-labeled *L. plantarum* ZP-6 over 24 h (Figure 5a). Fluorescence microscopy revealed: (i) 2 h post-administration: abundant green fluorescent foci appeared in the intestinal lumen, confirming rapid transit of viable bacteria; (ii) 4 h: fluorescence intensity peaked, indicating maximal bacterial colonization; (iii) 8 h: bacterial counts decreased significantly (*p* < 0.05 vs. 4 h), suggesting initial clearance; (iv) 12 h: only residual fluorescence was detectable, demonstrating near-complete evacuation. This biphasic colonization profile mirrored the temporal excretion pattern of fecal PS-NPs (Figures 1c,d), supporting a potential adsorption-clearance relationship. The synchronized decline in both bacterial counts and intestinal PS-NPs loads implies that *L. plantarum* ZP-6 may physically sequester NPs during transit, facilitating their elimination.

#### 3.5.2 Direct PS-NP adsorption by *L. plantarum* ZP-6

Building on our observation of enhanced fecal PS-NP excretion following *L. plantarum* ZP-6 intervention (Section 3.3.2), we hypothesized direct bacterial adsorption as the underlying mechanism. Confocal microscopy of fecal samples revealed: (i) Spatial co-localization: yellow-green fluorescence (*L. plantarum* ZP-6) and red fluorescence (PS-NPs) exhibited precise overlap (Figure 5b); (ii) Surface binding: PS-NPs adhered extensively to bacterial cell walls, forming distinct aggregates. These findings provide visual evidence that *L. plantarum* ZP-6: physically interacts with PS-NPs via surface adsorption, likely through electrostatic or hydrophobic interactions (given NP surface properties); functions as a biotic vector, transporting immobilized PS-NPs through the gastrointestinal tract for fecal excretion. So, these results revealed that the strain’s peptidoglycan layer or extracellular polysaccharides may serve as binding sites, consistent with prior reports of lactic acid bacteria adsorbing environmental pollutants; this “capture-and-remove” mechanism could explain the reduced systemic NPs accumulation observed in Section 3.3.

### 3.6 Mechanistic analysis of *L. plantarum* ZP-6 in alleviating PS-NP-induced liver damage via the gut-brain axis

#### 3.6.1 Analysis of intestinal mucus secretion

**Figure 6:**
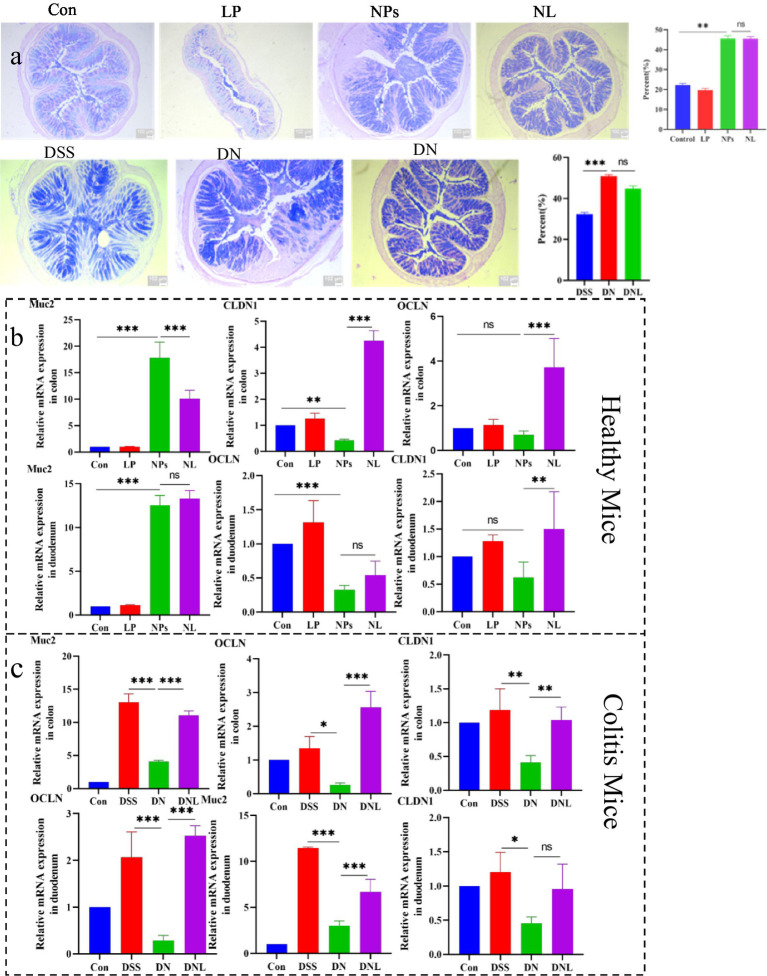
**(a)** Analysis of mucus secretion in mice colon. **(b)** Analysis of expression of mucin and intestinal barrier-related genes in healthy mice. **(c)** Analysis of expression of mucin and intestinal barrier-related genes in Colitis mice colon and duodenum. Con—control group; LP—*L. plantarum* group; NPs—nanoplastic group; NL—nanoplastic + *L. plantarum* group; DSS—acute colitis group; DN—colitis + nanoplastic group; DNL—colitis + nanoplastic + *L. plantarum* group; ns express *p* > 0.05, *p < 0.05, **p < 0.01, ***p < 0.001, *n* = 5.

The intestinal mucus layer constitutes a critical protective barrier against xenobiotic insults. Our investigation of mucus secretion patterns in PS-NPs-exposed mice revealed distinct responses in different physiological states (Figure 6). In healthy mice, PS-NPs exposure (NP_S_ group) induced a significant 2.3-fold (*p* < 0.01) increase in mucus coverage compared to controls (Figure 6a), suggesting an adaptive mucosal response to nanoparticle challenge.

Interestingly, similar hyper-secretion was observed in enteritis models, where PS-NPs-treated mice (DN group) exhibited 1.7-fold higher (*p* < 0.05) elevated mucus production relative to DSS-only controls. This conserved response across both healthy and inflamed intestines implies a fundamental defensive mechanism against PS-NPs intrusion. Notably, *L. plantarum* ZP-6 administration maintained this elevated mucus secretion without significant suppression, indicating its neutral effect on goblet cell secretory activity.

At the molecular level, we observed that *Muc2* gene expression mirrored these physiological changes (Figures 6b,c). In healthy mice, NP_S_ treatment upregulated *Muc2* transcription, 19.2-fold upregulation (*p* < 0.001) in colon and 10.06 increase in duodenum, consistent with the observed mucus hypersecretion. However, a paradoxical *Muc2* downregulation occurred in colitic mice following PS-NPs exposure, which was significantly reversed by *L. plantarum* ZP-6 treatment (DNL group), paradoxical about 30% suppression (*p* < 0.01) versus DSS controls. These differential responses suggest that PS-NPs may disrupt mucin homeostasis through distinct mechanisms depending on the inflammatory status of the intestine.

#### 3.6.2 Expression analysis of intestinal barrier-related factors

The integrity of intestinal tight junctions was systematically evaluated through quantification of *OCLN* and *CLDN1* gene expression (Figures 6b,c). PS-NPs exposure induced significant downregulation of these critical barrier proteins in both intestinal segments examined. In healthy mice, we observed tissue-specific suppression, with *CLDN1* most affected in the colon, 55% reduction in colonic *CLDN1* (*p* < 0.01); and *OCLN* in the duodenum, 64% decrease in duodenal *OCLN* (*p* < 0.01).

The compromise of barrier function was more pronounced in enteritis models, where PS-NPs exacerbated the DSS-induced downregulation of both genes, synergistic 60–80% downregulation of both genes (*p* < 0.001). This synergistic effect suggests that inflammatory conditions may potentiate PS-NPs toxicity to the intestinal epithelium. Importantly, *L. plantarum* ZP-6 treatment effectively restored tight junction protein expression in most tissues, though with varying efficacy depending on the specific gene and intestinal segment.

#### 3.6.3 Hepatic metabolomic analysis

**Figure 7:**
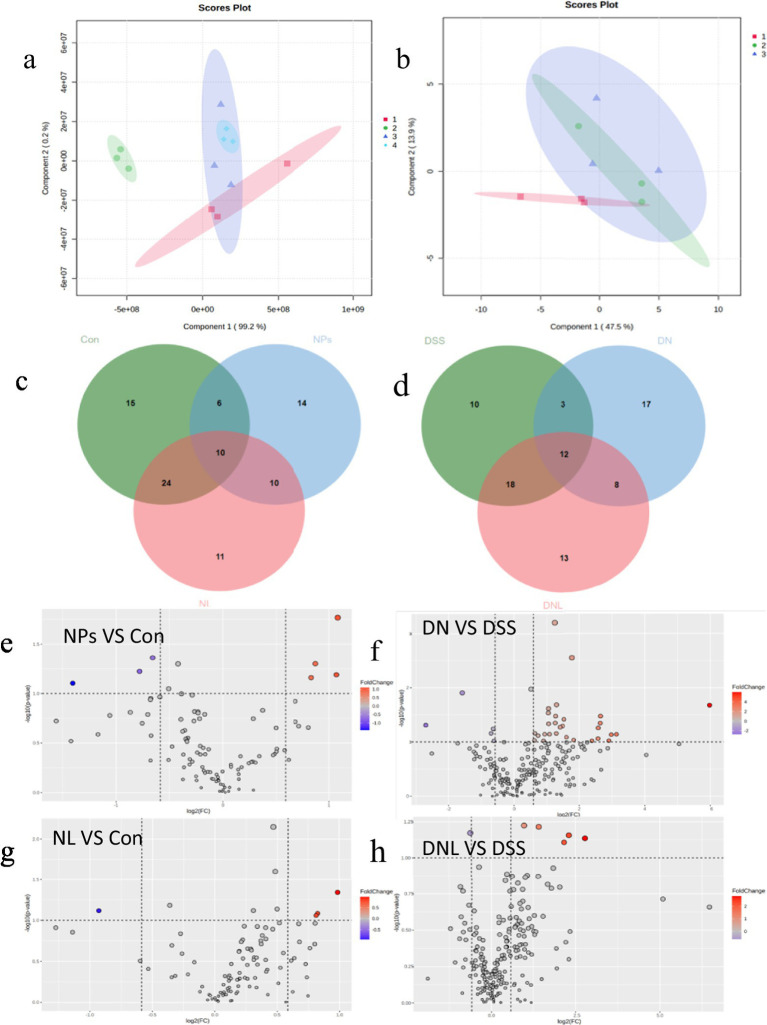
Analysis of mice liver metabolic profile. **(a,b)** Analysis of PLS-DA. **(a)** Healthy mice: 1-Con; 2-LP; 3-NL; 4-NPs. **(b)** Enteritis mice: 1-DSS; 2-DN; 3-DNL. **(c,d)** Venn diagram. **(c)** Healthy mice. **(d)** Colitis Mice. **(e–h)** Volcano plot of significantly different metabolite changes in mice liver. Con—control group; LP—*L. plantarum* group; NPs—nanoplastic group; NL—nanoplastic + *L. plantarum* group; DSS—acute colitis group; DN—colitis + nanoplastic group; DNL—colitis + nanoplastic + *L. plantarum* group.

PLS-DA of liver metabolic profiles revealed substantial perturbations induced by PS-NPs exposure (Figure 7). The metabolic fingerprints of NPs-treated groups diverged significantly from controls in both healthy and colitic mice, demonstrating systemic metabolic consequences of nanoparticle exposure.

Detailed analysis identified specific metabolic pathways affected: in healthy mice, 14 significantly altered metabolites, including depletion of stachyose and accumulation of guanosine and branched-chain amino acids, the notable changes include stachyose (−3.2-fold), guanosine (+2.8-fold); But in colitic mice, more extensive disturbances with 17 differential metabolites, featuring nucleotide metabolism alterations and widespread amino acid fluctuations, the key alterations include inosine (−4.1-fold), L-methionine (+3.5-fold).

*L. plantarum* ZP-6 intervention normalized many of these metabolic deviations, 78% reduction in metabolite alterations. Particularly restoring levels of key metabolites like nicotinamide (+2.4-fold) (with known anti-inflammatory properties) and sphingosine (+1.8-fold) (critical for membrane integrity). The partial overlap of confidence ellipses between treated and control groups indicates progressive metabolic recovery.

#### 3.6.4 Modulation of inflammatory responses

**Figure 8:**
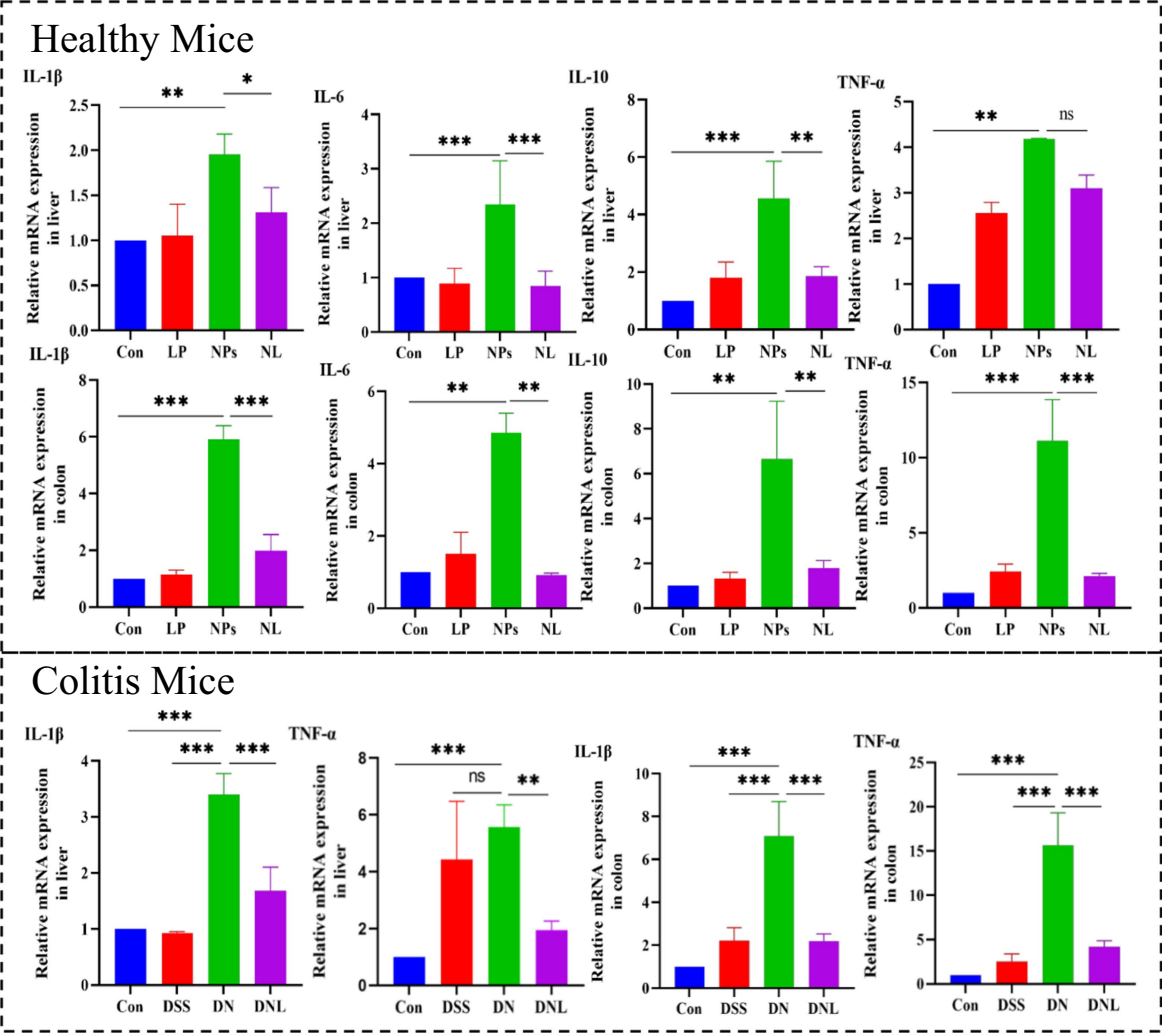
Expression of inflammatory factors in mouse liver and colon. Con—control group; LP—*L. plantarum* group; NPs—nanoplastic group; NL—nanoplastic + *L. plantarum* group; DSS—acute colitis group; DN—colitis + nanoplastic group; DNL—colitis + nanoplastic + *L. plantarum* group; ns express *p* > 0.05, *express *p* < 0.05, **express *p* < 0.01, ***express *p* < 0.001, *n* = 5.

**Figure 9:**
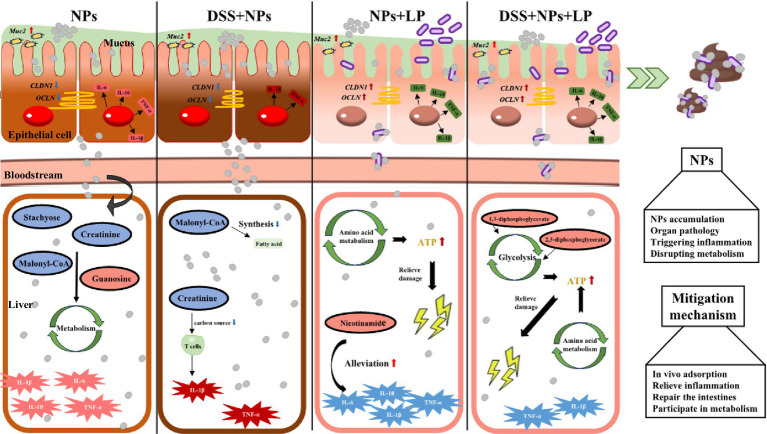
Liver damage induced by nanoplastics in mice with inflammatory bowel disease via the gut-liver axis and the alleviating effect of *L. plantarum* ZP-6. LP—*L. plantarum* group; NPs—nanoplastic group; NL—nanoplastic + *L. plantarum* group; DSS—acute colitis group; DN—colitis + nanoplastic group; DNL—colitis + nanoplastic + *L. plantarum* group.

PS-NPs exposure triggered robust inflammatory activation in both hepatic and colonic tissues (Figure 8). Pro-inflammatory cytokines (*IL-1β*, *IL-6*, *TNF-α*) were markedly elevated, *IL-1β* was 6.99-fold increase (*p* < 0.001) in colon and 1.9-fold elevation in liver (*p* < 0.01), *TNF-α* was 92.92-fold elevation (*p* < 0.001) in colon and 4.08-fold increase in liver (*p* < 0.01), IL-6 was 5.33-fold upregulation (*p* < 0.001) in colon and 2.29-fold increase in liver (*p* < 0.01), while the anti-inflammatory IL-10 showed more variable responses. This inflammatory signature was particularly pronounced in enteritis models, where PS-NPs amplified DSS-induced cytokine production. *L. plantarum* ZP-6 treatment consistently attenuated these inflammatory responses across tissues and disease states. The probiotic’s anti-inflammatory effects were most evident in colitic mice, where it significantly reduced *IL-1β* and *TNF-α* expression in both liver and colon, reduced *IL-1β* by 69% (*p* < 0.01) and normalized colonic *TNF-α* to baseline. Notably, this immunomodulation occurred in parallel with the observed improvements in barrier function and metabolic homeostasis, suggesting coordinated protection across multiple physiological systems (see Figure 9).

Collectively, our findings demonstrate that *L. plantarum* ZP-6 confers comprehensive protection against PS-NPs-induced toxicity through four synergistic mechanisms that collectively preserve gut-liver axis homeostasis. First, the probiotic orchestrates a sophisticated mucosal defense program, initiating rapid mucus hypersecretion followed by sustained Muc2-mediated regulation, with differential modulation observed between healthy and pathological conditions. Second, *L. plantarum* ZP-6 exhibits remarkable tissue specificity in intestinal barrier repair, differentially regulating *CLDN1* and *OCLN* expression to restore tight junction integrity and limit systemic nanoparticle translocation. Third, the strain mediates global metabolic reprogramming, effectively correcting PS-NPs-induced disturbances in nucleotide and amino acid metabolism while restoring critical metabolic pathways. Fourth, it demonstrates potent multi-organ anti-inflammatory activity, simultaneously attenuating pro-inflammatory signaling cascades in both hepatic and intestinal tissues. This multi-omics investigation positions *L. plantarum* ZP-6 as a master regulator of gut-brain-liver communication during nanotoxic stress, highlighting its potential as a novel biotherapeutic agent against environmental pollutant-induced damage. The coordinated nature of these protective mechanisms—spanning mucosal defense, barrier function, metabolic homeostasis, and inflammation control—underscores the unique capacity of specific probiotic strains to address complex toxicological challenges through microbiome-host crosstalk.

## 4 Discussion

The mechanisms underlying the hazards of NPs to biological systems are becoming increasingly elucidated. This study, utilizing a 5-week oral exposure protocol, confirmed that 100 nm PS-NPs penetrate the intestinal barrier of mice, enter the systemic circulation, and accumulate primarily in the liver and colon. This observation is highly consistent with the rapid entry into the bloodstream and organ distribution patterns of NPs reported by Im et al. (2022). Accumulated PS-NPs within the organism induced multi-organ damage: manifested as hepatocellular hypertrophy in the liver, mirroring the classic pathological feature of hepatocyte edema caused by MPs in previous studies (Zheng et al., 2021); accompanied by inflammatory cell infiltration in the kidney, aligning with descriptions of NPs-induced renal tubular necrosis in the study by Meng et al. (2023); and mechanical damage to the intestinal epithelium due to NPs aggregation and physical compression, paralleling the intestinal epithelial injury observed following NPs exposure in the study by Li et al. (2021). These pathological alterations were concomitant with a significant upregulation (*p* < 0.05) of pro-inflammatory cytokines *IL-1β*, *TNF-α*, and *IL-6*, establishing a systemic inflammatory cascade response. This reinforces the critical importance of pro-inflammatory cytokines as biomarkers for NPs exposure (Yu et al., 2018). Notably, the alterations in kidney coefficient and colon shortening identified in this study were not observed in the 28-day short-term exposure conducted by Xu et al. (2021). This discrepancy suggests that prolonged exposure (5 weeks) may trigger progressive, organ-specific damage, highlighting a significant concern for assessing the risks associated with chronic NPs exposure in humans.

Under pathological conditions, the toxicity of NPs exhibited a markedly amplified effect. When mice were in a state of DSS-induced colitis, PS-NPs exposure resulted in exacerbated hepatosplenomegaly, a significant increase in systemic NPs accumulation (approximately 3-fold higher than in healthy exposed mice), and a surge in the inflammatory cytokine *IL-1β* to levels approximately twice those observed in the healthy exposure group. This finding directly corroborates reports by Zheng et al. (2021) on NPs-aggravated hepatic injury in colitis and Luo et al. (2022) on NPs-promoted increases in intestinal permeability, collectively confirming that individuals with intestinal inflammation face a substantially heightened health threat from NPs.

To counteract these toxic effects, this study is the first to reveal that *L. plantarum* ZP-6 achieves potent intervention through multifaceted synergistic mechanisms. Firstly, *L. plantarum* ZP-6 effectively reduces systemic NPs accumulation via a bioadsorption-excretion pathway. ZP-6 facilitates the fecal excretion of adsorbed PS-NPs, leading to a 60–80% reduction (*p* < 0.01) in NPs burden within the liver. This mode of action is analogous to the mechanism reported by Chen et al. (2022), where lactic acid bacteria strains mitigate perfluorooctanesulfonic acid toxicity by promoting its excretion through bio-conjugation. Secondly, *L. plantarum* ZP-6 alleviates nanoplastic toxicity through an anti-inflammatory and barrier-repair pathway. It significantly suppressed the expression of hepatic pro-inflammatory cytokines (reducing *IL-1β* and *TNF-α* by 50–70%, *p* < 0.01), thereby disrupting the vicious cycle of inflammation, a finding consistent with the reported alleviation of intestinal and hepatic inflammation by *L. plantarum* in Zan et al. (2023). Furthermore, *L. plantarum* ZP-6 restored intestinal tight junction proteins, effectively reversing the PS-NPs-induced increase in intestinal permeability. This protective mechanism, akin to the restoration of ZO-1 protein and barrier integrity by *Lactobacillus casei* (Wu et al., 2024), successfully prevented secondary liver injury triggered by endotoxin translocation into the bloodstream (Denker and Nigam, 1998). Finally, at the metabolic level, *L. plantarum* ZP-6 demonstrated a corrective efficacy surpassing previous understanding. PS-NPs exposure profoundly disrupted hepatic metabolism in mice, including dysregulation of nucleotide metabolism (e.g., aberrant elevation of guanosine and significant reduction of inosine), imbalance in amino acid metabolism (e.g., accumulation of leucine/methionine), and impairment of fatty acid synthesis (e.g., decreased malonyl-CoA). These perturbations align with studies in zebrafish models showing PS-NPs interference with metabolic pathways (Liu et al., 2023; Limonta et al., 2019) and reports of NPs-induced metabolic dysregulation in mammals (Zheng et al., 2021). The unique value of *L. plantarum* ZP-6 lies in its ability to: upregulate the anti-inflammatory metabolite nicotinamide, known for its DNA repair functions (Mierzejewska et al., 2018); activate the glycolytic pathway (evidenced by significant increases in 1,3-bisphosphoglycerate), counteracting the energy depletion caused by NPs (Jiang et al., 2022); and reduce the number of significantly altered metabolites in colitic mice by 82%, effectively restoring the hepatic metabolic profile towards near-normal levels. This multi-targeted metabolic remodeling capability represents a more comprehensive homeostatic regulatory advantage compared to previous research on *L. plantarum* FRT10, which focused primarily on modulating amino acid or bile acid metabolism (Cai et al., 2022).

In conclusion, this study not only deepens the understanding of the toxicity associated with long-term exposure to small-sized PS-NPs (particularly the amplified risk in inflammatory bowel states) but also provides an innovative biological intervention strategy against nanoplastic pollution through the tripartite mechanism—“adsorption-excretion,” “anti-inflammatory repair,” and “metabolic remodeling”—exhibited by *L. plantarum* ZP-6. Compared to the singular toxin-adsorption function traditionally attributed to lactic acid bacteria (Chen et al., 2022), the multi-pathway synergy and metabolic corrective capacity of *L. plantarum* ZP-6 signify a crucial advancement in probiotic-based protective strategies.

## 5 Conclusion

This study demonstrates that exposure to PS-NPs exacerbates colonic and hepatic pathology in a murine colitis model, primarily mediated via the gut-liver axis. Crucially, *L. plantarum* ZP-6 was found to confer significant protection against PS-NP toxicity through a multi-targeted mechanism. The key findings reveal that ZP-6: (1) effectively sequesters PS-NPs directly, reducing systemic absorption and promoting fecal excretion; (2) modulates immune responses by significantly downregulating pro-inflammatory cytokines in both colon and liver, thereby restoring intestinal immune homeostasis, and interrupting the inflammation-fibrosis cycle; (3) strengthens the intestinal barrier through upregulation of key tight junction proteins, leading to substantial restoration of barrier integrity, reduced PS-NPs translocation; and (4) restores metabolic homeostasis. Collectively, this study offers a promising probiotic-based therapeutic strategy for mitigating nanoplastic risks in vulnerable populations (e.g., IBD patients). It identifies sensitive biomarkers for nanoplastic exposure assessment. Most importantly, it underscores the critical environmental health implication that pre-existing inflammation significantly amplifies nanoplastic toxicity, demanding urgent attention. While this study delineates compelling protective mechanisms for ZP-6, the findings are based on a mouse model of chemically-induced colitis, extrapolation to human IBD or other inflammatory conditions requires further validation. Future research should focus on conducting longitudinal studies to assess impacts on gut microbiome ecology and long-term health outcomes; and validating these findings in human-relevant models or cohorts.

## Data Availability

The original contributions presented in the study are included in the article/supplementary material, further inquiries can be directed to the corresponding authors.
